# Patient retention in antiretroviral therapy programs up to three years on treatment in sub-Saharan Africa, 2007–2009: systematic review

**DOI:** 10.1111/j.1365-3156.2010.02508.x

**Published:** 2010-06

**Authors:** Matthew P Fox, Sydney Rosen

**Affiliations:** 1 Center for Global Health and Development, Boston UniversityBoston MA, USA; 2 Health Economics and Epidemiology Research Office, Wits Health ConsortiumJohannesburg, South Africa; 3 Faculty of Health Sciences, University of the WitwatersrandJohannesburg, South Africa

**Keywords:** antiretroviral therapy, attrition, retention, sub-Saharan Africa, systematic review, human immuno-deficiency virus

## Abstract

**Objectives:**

To estimate the proportion of all-cause adult patient attrition from antiretroviral therapy (ART) programs in service delivery settings in sub-Saharan Africa through 36 months on treatment.

**Methods:**

We identified cohorts within Ovid Medline, ISI Web of Knowledge, Cochrane Database of Systematic Reviews and four conference abstract archives. We summarized retention rates from studies describing observational cohorts from sub-Saharan Africa reporting on adult HIV 1- infected patients initiating first-line three-drug ART. We estimated all-cause attrition rates for 6, 12, 18, 24, or 36 months after ART initiation including patients who died or were lost to follow-up (as defined by the author), but excluding transferred patients.

**Results:**

We analysed 33 sources describing 39 cohorts and 226 307 patients. Patients were more likely to be female (median 65%) and had a median age at initiation of 37 (range 34–40). Median starting CD4 count was 109 cells/mm^3^. Loss to follow-up was the most common cause of attrition (59%), followed by death (41%). Median attrition at 12, 24 and 36 months was 22.6% (range 7%–45%), 25% (range 11%–32%) and 29.5% (range 13%–36.1%) respectively. After pooling data in a random-effects meta-analysis, retention declined from 86.1% at 6 months to 80.2% at 12 months, 76.8% at 24 months and 72.3% at 36 months. Adjusting for variable follow-up time in a sensitivity analysis, 24 month retention was 70.0% (range: 66.7%–73.3%), while 36 month retention was 64.6% (range: 57.5%–72.1%).

**Conclusions:**

Our findings document the difficulties in retaining patients in care for lifelong treatment, and the progress being made in raising overall retention rates.

## Introduction

Although a great deal of research on daily adherence to antiretroviral therapy (ART) in sub-Saharan Africa has been published, long-term retention of patients in treatment programs has received comparatively less attention. ART has clearly been shown to be effective in reducing mortality among those who remain in treatment and adhere to therapy (Egger *et al.* 2002; Coetzee *et al.* 2004; Ivers *et al.* 2005; Laurent *et al.* 2005; Lawn *et al.* 2005), but under programmatic conditions, not all patients remain in treatment. In addition to known mortality while in care, some proportion of patients drop out of treatment programs and do not restart care elsewhere (Brinkhof *et al.* 2009; Fox *et al.* 2010). These patients are at high risk of morbidity and death within a short time. A valid measure for assessing the long-term success of ART programs should thus incorporate both mortality and loss to follow-up. In recent years, this measure has often been reported as the rate of retention, or the proportion of patients remaining alive and on ART at various time points after treatment initiation.

In 2007 we published the first systematic review of retention rates in cohorts in non-research settings in sub-Saharan Africa. In that review we demonstrated that, among over 74 000 patients representing 13 countries, the proportion of patients alive and on ART 2 years after initiation was approximately 60% (Rosen *et al.* 2007). The findings raised concern about high rates of attrition under programmatic conditions and suggested that more attention was needed to maintaining high rates of patient retention in both the months immediately following initiation when mortality is known to be high (Lawn *et al.* 2008), as well as over the whole course of patients’ lives.

Since 2007, a number of publications have reported retention experience in cohorts not included in the earlier systematic review or reported on a longer duration of follow-up of cohorts previously included. In this paper, we present an updated systematic review of information published between mid-2007 and mid-2009. We reviewed recent data on retention and estimated the rate of all-cause adult patient attrition from ART programs implemented in service delivery settings in sub-Saharan Africa to determine whether rates of retention have changed since our initial estimates and to extend the previous estimates, which covered 24 months of follow-up, through 36 months.

## Methods

To allow comparison between this review and the previous one, we maintained a similar methodology for this analysis as in our earlier review.

### Search strategy

The current analysis included studies describing the results of observational cohort data from sub-Saharan Africa which reported on all adult HIV 1- infected patients who initiated first-line three drug combination ART, including those who discontinued treatment for any reason. We included cohorts from any facility, whether public, nongovernmental, or private, as long as they treated the general population using standard therapy. We excluded clinical trials and clinics serving specialized populations, such as workforces. To avoid double-counting, we excluded reports of pooled data where it appeared that there was substantial overlap with patients also included in single-cohort reports. We required information beginning at ART initiation and a minimum, mean, or median follow-up of 6 full months (26 weeks). In some cases the average follow-up was not clearly specified but it could either be calculated from information provided or determined that it must be greater than 6 months. In cases where a cohort reported >50% retention at the last time point reported to, but no median duration of follow-up, the last time point reported to was used as the median. Reports had to include all-cause attrition rates for at least one of 6, 12, 18, 24, or 36 months after ART initiation. Studies that did not report on these time points but did provide enough information to calculate one of these rates were also included. When possible, children and non-naïve patients were excluded.

To identify studies for the current analysis, we searched Ovid Medline 2007-week 2, August 2009, ISI Web of Knowledge 2007-August 26, 2009 and Cochrane Database of Systematic Reviews 2nd quarter 2009) and four conference abstract archives (Conference on Retroviruses and Opportunistic Infections 2008–2009 International AIDS Conference 2007–2009, HIV Implementers Meeting 2008–2009 and International Association of Physicians in AIDS Care 3rd Conference on Treatment Adherence). The search in Medline, ISI, and the Cochrane Database combined the terms ‘antiretroviral’ and ‘Africa’ with any one of the following: retention/attrition/adherence/mortality/loss to follow-up/efficacy/evaluation or the term ‘antiretroviral’ and ‘developing country’ with either ‘adherence’ or ‘mortality’. Conference abstracts were searched for any of the terms ‘attrition’‘retention’ or ‘lost to follow-up’ except for the IAPAC conference in which all abstracts were scanned.

SR identified the eligibility of all abstracts and journal articles that met our initial search terms and MF confirmed eligibility. For each study identified for inclusion we used a standard data extraction form to collect the relevant data. In cases where multiple reports described the same cohort, the one reporting to the longest time point or with the most complete information was used.

### Definitions

We defined attrition from ART programs to include patients who died or were lost to follow-up. We defined retention to be the opposite of attrition (i.e. 1 – attrition). As reporting of patients who were still in care but had stopped taking ARVs was variable, we did not exclude these patients from the total retained in care. We accepted the varying definitions used in the reports for loss to follow-up and provide these definitions in Table 1. We excluded patients who were transferred to another facility from both the numerator and denominator of calculations of retention as we could not assess their outcomes.

**Table 1:** Characteristics of reports, cohorts, and patients included in this review of attrition rates from ART Programs in sub-Saharan Africa

Study Code	Reference	Country	Eligibility criteria (; = ‘or’)*	LTFU definition	Facilities (*n*)	Sector	Payment by patient required?	Dates of cohort observation	Cohort size (*n*)	Median age (years)	Female (%)	Median starting CD4 count (Cells/mm^3^)
Botswana 1 (Bisson *et al.* 2008)	Bisson 2008	Botswana	CD4 < 200; OI	30 days late for last scheduled appt	1	Public	No	Feb 2003 – Mar 2004	410	37†	60	81
Botswana 2 (Bussmann *et al.* 2008)	Bussman 2008	Botswana	CD4 < 200; ADOI	Not reported	1	Public	No	Jan 2002 – Apr 2007	633	35	60	67
Cameroon 1 (Sieleunou *et al.* 2009)	Sieleunou 2009	Cameroon‡	CD4 < 200; WHO 3 or 4	>3 months late for last visit	1	NGO	Partial	Jul 2001 – Jun 2007	1,187	35	44	105
Congo 1 (O’Brien *et al.* 2009)	O’Brien 2009	Congo§	‘WHO recommendations’ (cites 2006 WHO guidelines)	Not reported	2	NGO	No	Mar 2005 – Dec 2007	222	37	69	104
Cote d’Ivoire1 (Toure *et al.* 2008)	Toure 2008	Cote d’Ivoire	CD4 < 200; WHO 4; WHO 3 + CD4 200–350	>3 months since last contact with clinic	Multiple	NGO	Partial	May 2004 – Feb 2007	10,211	36	70	123
DRC 1 (Culbert *et al.* 2007)	Culbert 2007	DRC Congo	‘WHO guidelines’ (cites 2003 guidelines)	Not reported	2	NGO	No	Oct 2003 – Jan 2006¶	494	37	66	123
Ethiopia 1 (Gugsa 2008)	Gugsa 2008	Ethiopia	Not reported	Not reported	1	Public	–	Sep 2005 – Sep 2006¶	321	–	59	102**
Ghana 1 (Chiliade 2008)	Chiliade 2008	Ghana	Not reported	Not reported	Multiple	NGO	–	–	5,844	–	63	114
Ghana 2 (Collini *et al.* 2009)	Collini 2009	Ghana‡	CD4 < 250; WHO 3 or 4	Not reported	1	Public	Partial	Jan 2004 –	237	40 †	59	120†
Kenya 1 (Unge *et al.* 2009)	Unge 2009	Kenya‡	WHO stage (not specified)	> 90 days after last prescribed dose	1	NGO	Free	Jan 2005 – Sep 2007¶	830	35	65	203
Malawi 1 (Jahn 2008)	Jahn 2008	Malawi††	Not reported	Not reported	146	Public	–	Jun 2004 – Jul 2007¶	114,375	–	61	–
Mozambique 1 (Auld 2009)	Auld 2009	Mozambique‡‡	Not reported	Not reported	30	Public	–	2004 – 2007¶	2,596	34	62	–
Multiple 1 (Marazzi *et al.* 2008)	Marazzi 2008	Mozambique, Tanzania, Malawi	CD4 < 200 + WHO 3 or 4; CD4 200–350 + viral load >55,000	Not reported	12	NGO	–	Jan 2003 – Jun 2006	3,456	37	60	166†
Multiple 2 (Palombi *et al.* 2009)	Palombi 2009	Mozambique, Malawi, Guinea‡	Not reported	>3 months since last contact	5	NGO	–	–Jun 2007	3,749	34	62	192
Nigeria 1 (DeSilva *et al.* 2009)	DeSilva 2009	Nigeria	Not reported	No visit for > 3 months before data collection	1	NGO	–	Jan 2005 – Dec 2006	1,552	34	71	112
Rwanda 1 (Chiliade 2008)	Chiliade 2008	Rwanda	Not reported	Not reported	Multiple	NGO	–	–	1,707	–	64	150
Rwanda 2 (Lowrance *et al.* 2009)	Lowrance 2009	Rwanda‡‡	WHO 4; WHO 3 + CD4 < 350; WHO 1 or 2 + CD4 < 200	>90 days since last contact	30	Public	–	Jan 2004 – Dec 2005	3,194	37	65	141
SA 1 (Boulle *et al.* 2008)	Boulle 2008	South Africa	WHO 4 excluding EPTB; CD4 < 200	>90 days since last contact	Multiple	Public	–	May 2001 – Mar 2006	12,587	–	70	–
SA 2 (Barth *et al.* 2009)	Barth 2008	South Africa	Not reported	Not reported	1	NGO	–	–	735	34	72	68
SA 3 (Dahab 2008)	Dahab 2008	South Africa	Not reported	>1 month late for six–month visit	1	Public	No	–	267	37	67	–
SA 4a (Grimwood A 2008)	Grimwood 2008	South Africa	Not reported	Not reported	–	Public	No	Jan 2003 – Dec 2007¶	6,469	–	–	117
SA 4b (Grimwood A 2008)	Grimwood 2008	South Africa	Not reported	Not reported	–	Public	No	Jan 2003 – Dec 2007¶	1,135	–	–	127
SA 5 (Khan 2009)	Khan 2009	South Africa§§	Not reported	‘Failure to collect ARV’	1	Public	–	Jul 2004 – Jun 2005¶	684	–	73	–
SA 6 (Kaplan *et al.* 2008)	Kaplan 2008	South Africa¶¶	Not reported	No clinic visit > 12 weeks	1	Public	No	Sep 2002 – Sep 2007¶	1,677	–	100	–
SA 7 (MacPherson *et al.* 2009)	MacPherson 2009	South Africa	CD4 < 200 + WHO 4 + ‘psychosocial preparedness to undertake therapy’	>1 day late for appointment, could not be traced, and did not come back during study period	1	Public	No	Oct 2005 – Sept 2007¶	1,353	37	67	93
SA 8 (Nachega *et al.* 2008)	Nachega 2008	South Africa***	Two CD4s<350; confirmed ADOI	Leaving medical insurance fund or AID for AIDS program	Multiple	Private	Yes	Jan 1998 – Sep 2004¶	2,817	37†	63	147
SA 9 (Ojikutu *et al.* 2008)	Ojikutu 2008	South Africa†††	CD4 < 200; WHO 4	No clinic visit within 6 months of end of study	1	NGO	Yes	Jan 1999 – Feb 2004¶	309	38†	56	65
SA 10a (Rosen *et al.* 2008)	Rosen 2008	South Africa	CD4 < 200	>3 months late for last visit	1	Public	Partial	Jan 2005 – Dec 2006	100	–	–	97‡‡‡
SA 10b (Rosen *et al.* 2008)	Rosen 2008	South Africa	CD4 < 200	>3 months late for last visit	1	Private	No	Jan 2005 – Dec 2006	100	–	–	84‡‡‡
SA 10c (Rosen *et al.* 2008)	Rosen 2008	South Africa	CD4 < 200	>3 months late for last visit	1	NGO	Partial	Jan 2005 – Dec 2006	100	–	–	60‡‡‡
SA 10d (Rosen *et al.* 2008)	Rosen 2008	South Africa	CD4 < 200	>3 months late for last visit	1	NGO	Partial	Jan 2005 – Dec 2006	100	–	–	104‡‡‡
Tanzania 1 (Chalamilla 2008)	Chalamilla 2008	Tanzania	Not reported	Not reported	–	–	–	Nov 2004 – Apr 2007¶	6,893	37	71	133
Tanzania 2 (Johannessen *et al.* 2008)	Johannessen 2008	Tanzania	CD4 < 200; WHO 4; WHO 3 + CD4 200–350	>3 months late for last appt and could not be traced	1	NGO	No	Oct 2003 – May 2007	320	35	70	–
Uganda 1a (Ahoua *et al.* 2009)	Ahoua 2009	Uganda	WHO guidelines (cites 2003 guidelines)	>2 months late for last appt	1	Public	No	Sept 2004 – May 2006	967	37	65	100
Uganda 1b (Ahoua *et al.* 2009)	Ahoua 2009	Uganda	WHO guidelines (cites 2003 guidelines)	>2 months late for last appt	1	Public	No	Sept 2003 – May 2006	556	37	62	93
Uganda 2 (Chang *et al.* 2009)	Chang 2009	Uganda	CD4 < 250; WHO 3 or 4	>90 days since last visit and on ART	1	NGO	No	Oct 2003 – Apr 2006	360	38§§§	66§§§	100§§§
Uganda 3 (Bajunirwe *et al.* 2007)	Bajunirwe 2007	Uganda	Not reported	Not reported	1	Public	–	Dec 2004 – Dec 2006¶	398	–	–	–
Uganda 4 (Martin 2008)	Martin 2008	Uganda	Not reported	‘Where abouts unknown for 6 months or more’	1	–	No	–	323	35	71	124
Zambia 1 (Chi *et al.* 2009)	Chi 2009	Zambia	CD4 < 200; WHO 4; WHO 3 + CD4 200–350	>30 days late for last medication pickup and can not be traced	18	Public	No	Apr 2004 – Nov 2008	37,039	35¶¶¶	61¶¶¶	126¶¶¶
Total or weighted average									226,307****	36††††	63††††	128††††

“—” indicates information not reported in study.

NGO, nongovernmental organization, ADOI, AIDS defining opportunistic infection, EPTB, extra-pulmonary tuberculosis.

* Many public sector ART programs that did not report eligibility criteria likely followed national guidelines, which are typically consistent with WHO recommendations.

† Median not reported, mean reported instead.

‡ Required at least one follow-up visit.

§ Includes non-naïve patients.

¶ Period during which study participants initiated ART; follow-up extends beyond dates shown.

** Weighted the medians for those lost, died or retained by sample size.

†† Sampled entire national program (adults).

‡‡ Representative sample of the national program.

§§ Includes 75 children.

¶¶ Rate from KM estimates only. Weighted average of pregnant and non-pregnant subjects.

*** Study compared EFV and NVP; rates shown are for combined cohort.

††† Includes non-naïve patients. Patients with missing records excluded.

‡‡‡ Weighted average of outcome groups.

§§§ Weighted averages of those on treatment and those not on treatment.

¶¶¶ Weighted averages of those on treatment at 12 months and those not.

**** Total.

†††† Weighted by sample size.

### Statistical methods

We first described each cohort and summarized its demographic and clinical characteristics by weighting the reported values by the sample size. We plotted the reported crude retention rates from each study at up to five time points: 6, 12, 18, 24 and 36 months. When retention rates were reported at time points different from those listed above or only for the median duration of follow-up, we applied the reported rates to the time point to which they were closest. For each study we calculated simple retention proportions (%RT) at each time point t as:

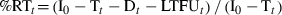

where I_0_ is all patients initiated on ART at the site; T_t_ is all patients transferred out of care by time t; D_t_ is all patients who died by time t; and LTFU_t_ is all patients lost to follow-up by time t. This is the proportion of all patients initiated who did not transfer out of care who are still alive and in care at the end of the follow-up period.

In cases where the first time point for which a study reported retention rates was later than 6 months after treatment initiation (25 studies representing 42 observations) we imputed the earlier missing time points. Because rates of attrition are unlikely to be constant over time (i.e. a linear decline), but rather likely to show a sharp decline over the first 6-months in care and level out sometime after 1 year, we first fit a linear random-effects regression model using all the data available on current retention as a function of time, time squared, and time cubed with a random intercept for each study. The cubic form had a better fit than linear, quadratic, or half root forms. We then took the resulting predicted curve and calculated the proportion of the total 36-month attrition that was expected to have occurred by each time period, with the constraint that retention could not increase over time. In this model, of total retention by 36 months, cumulatively 56% occurred by 6 months, 83% occurred by 12 months, 91% occurred by 18 months, 96% occurred by 24 months and 100% occurred by 36 months. This information was used to calculate the % retention at earlier missing time points as:

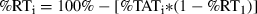

where %RT_i_ is the missing retention rate at month t_i_, 100% and %RT_1_ are the two reported retention rates at baseline and time t_1_ that bound %RT_i_ (i.e. 0 < t_i_ < t_1_), and %TAT_i_ is the % of total attrition up to the first time period reported (i.e. %RT_1_) that occurred by time t_i_. For example, if the first retention proportion reported was 80% at 12 months, the percent of total attrition up to 12 months that occurred by 6 months would be:

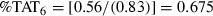

meaning that 67.5% of the total 20% attrition by 12 months occurred by 6-months. We would then estimate the 6- month retention proportion to be:

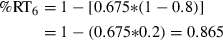


For all time periods after the first reported retention proportion, if at least two non-consecutive time points of interest were reported but the study did not give retention proportions at one or more of the time points in between, we interpolated proportions (%RT_i_) for the missing time points as a linear decline:

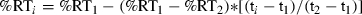

where %RT_i_ is the interpolated retention proportion at month t_i_ and %RT_1_ and %RT_2_ are the two reported retention proportions at months t_1_ and t_2_ that bound %RT_i_ (i.e. t_1_ < t_i_ < t_2_). This was carried out for seven of the studies representing eight imputations.

As we previously demonstrated a relationship between duration of follow-up and reported retention rates (Rosen *et al.* 2007) (e.g. studies reporting only 6 months of follow-up on average reported lower retention at 6 months than the reported 6 month retention in studies reporting to 12 or 24 months), we first described retention rates in the current dataset by plotting them over time grouped by duration of reporting. We display retention rates at 6, 12, 24 and 36 months using forest plots to allow for visualization of the distribution of retention rates at each time point. We then summarized these rates using random-effects meta-analysis (Egger *et al.* 2001) using a Freeman & Tukey (1950) arc-sin transformation of the retention proportions and standard errors and presented point estimates of retention and corresponding 95% confidence intervals.

To explore the impact of duration of reporting on overall retention, we projected the path of retention rates by extrapolating retention rates through 3 years (36 months) for all studies from their time of last reporting using three methods. We calculated a best-case scenario using last observation carried forward from the last time point reported through 3 years. We calculated a worst-case scenario by assuming that the attrition rates continued along the linear slope calculated between the first and last time point reported, with retention truncated at 0%. We calculated a midpoint scenario as the average of the two. In all scenarios, results were weighted by cohort size.

Finally, we looked for predictors of retention using linear regression. Potential predictors included median age in years (<36 *vs.*≥36), % female (<60%*vs.*≥60%), median baseline CD4 count (<100 *vs.*≥100), duration of follow-up (≤12 months *vs.* >12 months), patient payment required (yes *vs.* no), sector (public/private/other), and year of initiation of the cohort (<2004 *vs.*≥2004). Because we had only 39 observations, all missing data were coded as unknown and included in regression analyses, but inferences were only drawn comparing the groups for which we had data.

Two other potential, program-level predictors of retention are whether or not the treatment provider undertook active tracing of patients who were lost and made attempts to return them to care and how much pre-ART counseling was given to prepare patients for initiating and maintaining ART. Practices for preparing patients for ART and tracing those lost to follow up were not consistently reported, however, and therefore could not be included in the current analysis.

## Results

**Figure 1:**
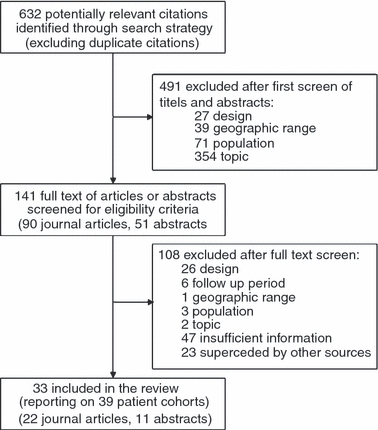
Literature Search and Application of Eligibility Criteria in a Systematic Review of Retention in Antiretroviral Therapy Programs in Sub-Saharan Africa.

Our search identified 632 papers and abstracts after removing duplicates. Of these, 491 were excluded on the basis of title or abstract, and an additional 108 were excluded after a full text screen and removal of overlapping cohorts and reports that contained insufficient information. Thirty-three sources remained that met our eligibility criteria (Figure 1), including 22 full text journal articles and 11 abstracts. These described outcomes for a total of 39 cohorts and 226 307 patients.

Several exclusions from the review and censoring of data should be noted. First, we omitted reports from the International epidemiological Databases to Evaluate AIDS (IeDEA) group that pools data from HIV/AIDS treatment programs. We excluded these reports because many of the cohorts included in IeDEA’s analyses are described in more detail in the individual papers and abstracts in our review. Second, two studies, SA 1 and SA 2, reported outcomes to 48 months, and two studies, Botswana 2 and Cameroon 1, reported outcomes to 60 months. As these were the only cohorts reporting to time periods beyond 36 months, we censored data from these cohorts at the last time period reported to before 60 months (12 months for Cameroon 1 and 36 months for the remaining 3). And finally, we excluded several publications reporting data for cohorts from Malawi because the most recent report for Malawi, a conference abstract from July 2009 (Malawi 1), included data for the entire Malawian national ART program, thus encompassing the cohorts described in the other papers.

Table 1 describes the characteristics of the cohorts. Nearly half the cohorts (49%, 19/39), representing 81% of the patients (183,486/226,307), were in southern Africa. The majority of treatment sites were either public sector (53%) or NGO (41%) facilities. About 35% of the 25 cohorts that provided information on payment reported that patients had to pay to receive care. The median years in which cohorts were enrolled for observation and in which observation ended were 2004 (range 1998–2005) and 2006 (range 2002–2007).

Table 1 also reports median patient characteristics for each cohort. Patients were more likely to be female (median 65%, range 44%–100%) and had a median age at initiation of ART of 37 (range 34–40). All cohorts that reported starting CD4 count except one (Kenya 1) had a median below 200 cells/mm^3^ (median 113, range 60–203). For the studies that reported it, the median of the reported follow-up times was 12 months.

The proportion of patients lost from each cohort at the end of that cohort’s follow-up stratified by the cause of attrition, as well as the total proportion retained at each site, is shown in Table 2. After weighting for cohort size, loss to follow-up (LTFU) was the most common cause of attrition, followed by death (59% and 41% of total attrition respectively).

**Table 2:** Rates of Patient Attrition and Retention from Antiretroviral Treatment Programs in Sub-Saharan Africa, as reported

Study Code	Median follow-up (months)	Died (A)	Lost to follow-up (B)	Total attrition from ART (C)*	Total retained (D)*	Transferred care (E)	Total retained at original site (F)*
Botswana 1†	10.1	16.8%	5.4%	22.2%	77.8%	0.0%	77.8%
Botswana 2	41.9	19.0%	16.1%	35.1%	64.9%	19.1%	45.8%
Cameroon 1	49.0	28.5%	5.0%	33.4%	66.6%	18.5%	48.0%
Congo 1	9.0¶	9.0%	13.1%	22.1%	77.9%	0.0%	77.9%
Cote d’Ivoire 1	7.7	11.2%	13.6%	24.7%	75.3%	3.0%	72.3%
DRC 1	6.0‡	7.9%	5.5%	13.4%	86.6%	0.0%	86.6%
Ethiopia 1	6.0‡	18.4%	8.1%	26.5%	73.5%	1.3%	72.2%
Ghana 1	24.0‡	–	–	–	–	–	–
Ghana 2	36.0‡	0.0%	30.8%	30.8%	69.2%	3.4%	65.8%
Kenya 1	15.2§	0.0%	29.4%	29.4%	70.6%	0.0%	70.6%
Malawi 1	36.0‡	–	–	–	–	–	–
Mozambique 1	12.0‡	5.0%	15.0%	20.0%	80.0%	0.0%	80.0%
Multiple 1	12.0	7.5%	1.2%	8.7%	91.3%	0.0%	91.3%
Multiple 2	14.5¶	10.5%	2.8%	13.3%	86.7%	0.0%	86.7%
Nigeria 1	14.6¶	6.7%	8.8%	15.5%	84.5%	0.0%	84.5%
Rwanda 1	24.0‡	–	–	–	–	–	–
Rwanda 2	14.9¶	4.3%	4.5%	8.8%	91.2%	4.0%	87.2%
SA 1	–**	–	–	–	–	–	–
SA 2	37.0¶	23.3%	12.0%	35.2%	64.8%	6.4%	58.4%
SA 3	6.0‡	6.7%	8.6%	15.4%	84.6%	1.1%	83.5%
SA 4a	24.0‡	9.0%	11.4%	20.4%	79.6%	0.0%	79.6%
SA 4b	36.0‡	6.0%	7.0%	13.0%	87.0%	0.0%	87.0%
SA 5	36.0‡	18.0%	5.3%	23.2%	76.8%	3.9%	72.8%
SA 6	–**	–	–	–	–	–	–
SA 7	8.6¶	9.2%	2.6%	11.8%	88.2%	4.7%	83.6%
SA 8	24.0	2.0%	11.2%	13.2%	86.8%	0.0%	86.8%
SA 9	8.4¶	15.9%	7.4%	23.3%	76.7%	0.0%	76.7%
SA 10a	12.0	2.1%	23.9%	26.0%	74.0%	0.0%	74.0%
SA 10b	12.0	18.9%	26.1%	45.0%	55.0%	0.0%	55.0%
SA 10c	12.0	12.9%	15.1%	28.0%	72.0%	0.0%	72.0%
SA 10d	12.0	7.0%	6.0%	13.0%	87.0%	0.0%	87.0%
Tanzania 1	7.9¶	2.5%	12.1%	14.6%	85.4%	0.0%	85.4%
Tanzania 2	10.9	29.7%	9.7%	39.4%	60.6%	10.9%	49.7%
Uganda 1a	12.0‡	5.0%	19.3%	24.3%	75.7%	0.0%	75.7%
Uganda 1b	24.0‡	14.7%	17.1%	31.8%	68.2%	0.0%	68.2%
Uganda 2	24.0‡	18.3%	7.8%	26.1%	73.9%	0.6%	73.3%
Uganda 3	–**	–	–	–	–	–	–
Uganda 4	8.3	5.0%††	4.0%	9.0%	91.0%	0.0%	91.0%
Zambia 1	12.0‡	9.9%	16.9%	26.8%	73.2%	0.0%	73.2%
Simple averages	18.3	10.9%	11.6%	22.5%	77.5%	2.3%	75.1%
Weighted averages‡‡	26.3	9.1%	13.0%	22.1%	77.9%	1.1%	76.8%

“—” indicates that these data could not be determined from the report.

* Calculations: C = A+B; D = 1 – C; F = D–E.

† Rates are based on results after active tracing of patients.

‡ Median not reported but estimated as last time period reported to as >50% of cohort was still retained.

§ Median not reported; weighted mean for those lost and those retained by sample size.

¶ Median not reported; table shows mean follow-up instead.

** Not reported but > 6 months.

†† Estimated from KM curve.

‡‡ Weighted by cohort size.

Total retention rates at each time point reported are presented in Table 3. For the 39 cohorts, attrition rates were reported at only one time point for 24 (61%); the median time point for these cohorts was 18 months (IQR 12–24 months). Total attrition at 12 months was quite variable, with a median of 22.6% and a range from 7 to 45% (Rwanda 1 and SA 10b respectively). There was little change in median attrition (27%) by 24 months, but the range narrowed slightly, from 11% to 35% in Rwanda 1 and Kenya 1 respectively. By 36 months median attrition increased to 29.6%, with estimates ranging from 13.0% to 36.1% (in SA 4b and Botswana 2 respectively).

**Table 3:** Retention of Patients at 6, 12, 18, 24 and 36 months after initiation of antiretroviral therapy in sub-Saharan Africa

	Percentage of patients retained at month					
	
Study code	6	12	18	24	36	Notes
Botswana 1	–	77.8%	–	–	–	12 month value is at a median of 10.1 months
Botswana 2	–	73.8%	–	–	63.9%	Values estimated from KM estimates of LTFU and death
Cameroon 1	–	65.0%	–	–	–	Values estimated from KM curves counting LTFU as an event
Congo 1	94.0%	89.0%	–	–	–	Survival reported as combined LTFU and death
Cote d’Ivoire 1	–	–	64.0%	–	–	18 month estimate of death and LTFU summed
DRC 1	–	86.7%	–	–	–	
Ethiopia 1	72.3%	–	–	–	–	
Ghana 1	87.0%	81.0%	77.0%	68.0%	–	
Ghana 2	–	82.5%	74.7%	–	68.1%	Estimated based on number of patients reporting for follow-up visits; no deaths reported
Kenya 1	83.0%	74.0%	–	65.0%	–	Estimated using survival analysis. No deaths reported
Malawi 1	–	77.0%	–	70.0%	68.0%	Includes stopping ART as attrition
Mozambique 1	86.0%	79.0%	–	–	–	
Multiple 1	–	91.3%	–	–	–	Attributed total death and LTFU to 12 months
Multiple 2	–	–	86.7%	–	–	18 months value based on mean 15 months follow-up
Nigeria 1	–	–	84.5%	–	–	18 months value based on mean 15 months follow-up
Rwanda 1	94.0%	93.0%	91.0%	89.0%	–	
Rwanda 2	93.0%	91.0%	–	–	–	Excluded transfers and stopping ART from reported attrition
SA 1	89.1%	84.9%	81.3%	80.9%	77.0%	Estimates are from nested cohorts
SA 2	76.0%	71.0%	70.0%	68.0%	64.0%	Estimated from KM curves; includes patients transferred as attrition
SA 3	83.5%	–	–	–	–	
SA 4a	–	–	–	79.6%	–	
SA 4b	–	–	–	–	87.0%	
SA 5	87.4%	84.1%	–	77.6%	72.8%	Includes patients transferred as attrition
SA 6	–	–	–	–	74.3%	Rate from KM estimates. Weighted average of pregnant and non-pregnant subjects.
SA 7	–	–	–	83.6%	–	
SA 8	–	–	–	86.8%	–	Study compared EFV and NVP; rates shown are for combined cohort.
SA 9	76.7%	–	–	–	–	6 month estimate at 8 months
SA 10a	–	74.0%	–	–	–	
SA 10b	–	55.0%	–	–	–	
SA 10c	–	72.0%	–	–	–	
SA 10d	–	87.0%	–	–	–	
Tanzania 1	–	85.4%	–	–	–	
Tanzania 2	–	60.6%	–	–	–	12 month estimate at 11 months
Uganda 1a	–	75.7%	–	–	–	Data are from retrospective cohort analysis; excluded transfers and stopping ART from reported attrition
Uganda 1b	–	–	–	68.2%	–	Data is from retrospective cohort analysis; excluded transfers and stopping ART from reported attrition
Uganda 2	85.0%	77.0%	–	73.0%	–	Includes those who stopped treatment
Uganda 3	–	76.0%	–	71.0%	–	
Uganda 4	91.0%	–	–	–	–	6 month estimate at 8.3 months
Zambia 1	–	73.2%	–	–	–	
Simple averages	85.6%	78.3%	78.6%	75.4%	71.9%	

KM, Kaplan-Meier.

To account for the variable times reported to, in all analyses presented below we interpolated retention rates at any time point where an estimate of retention was missing but a later time point was reported. Fifty estimates of attrition were interpolated, most at 6, 12, or 18 months (*n* = 25, 9, and 11, respectively).

**Figure 2:**
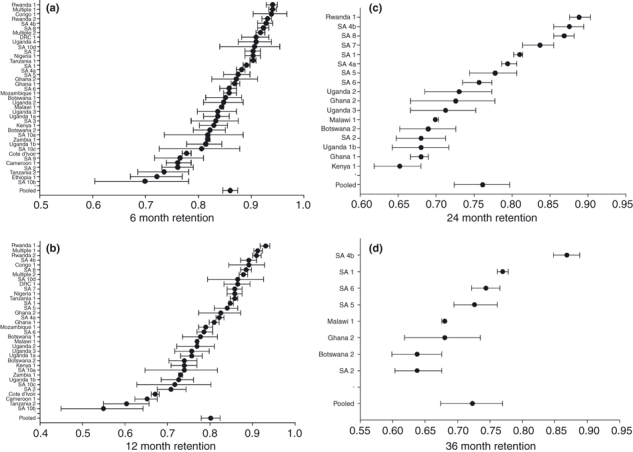
a–d Forest Plots of Reported Retention at 6, 12, 24 and 36 Months after Initiation of Antiretroviral Therapy in Sub-Saharan Africa*. *Pooled estimates were created using random-effects meta-analysis. Data include both actual reported rates for studies that reported to each time point and linear interpolation for studies which reported to a later time point but not the current time point.

Figure 2a–d show the variation in retention rates at 6, 12, 24 and 36 months using forest plots. Using random effects meta-analysis to pool the data including the interpolated time points, we estimated the retention at 6 months to be 86.1% (95% CI: 84.6%–87.4%), at 12 months to be 80.2% (95% CI: 78.0%–82.4%), at 24 months to be 76.1% (95% CI: 72.4%–79.7%) and at 36 months to be 72.3% (95% CI: 67.4%–76.9%).

**Figure 3:**
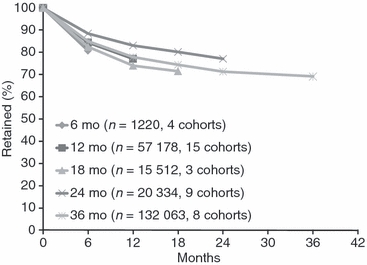
Weighted Average Retention Rates Over Time in Antiretroviral Therapy Programs in Sub-Saharan Africa*. *Studies reporting later time points and not earlier ones had the earlier attrition rates interpolated as described in the methods section and then weighted by cohort size.

We were concerned that studies reporting only to shorter time points would have higher attrition at those time points than would studies that reported longer follow-up. Figure 3 shows retention rates stratified by last reported time point. Each time point shows variation in retention rates, but there is no clear picture of studies reporting at later time points having higher overall retention at earlier time points compared to studies reporting only to earlier time points. While the cohorts reporting only to 6 and 12 months show lower attrition with duration of time reported to, suggesting some bias may exist, the 8 cohorts reporting to 36 months show sharper declines in the first 6–12 months than cohorts reporting to 24 months.

Using linear regression we found that median starting CD4 count <100 (−5.8%; 95% CI: −8.9% to −2.7%), median age <36 (5.7%; 95% CI: −9.2% to −2.2%), and having <60% females (−9.4%; 95% CI: −13.9% to −4.9%) were predictive of lower retention rates at 6 months when also adjusting for median follow-up and year of initiating cohort. For 12 month attrition rates, only median age <36 (−12.8%; 95% CI: −19.9% to −5.6%), median CD4 < 100 (−8.8%; 95% CI: −14.5% to −3.1%) and cohort follow-up ≤12 months (−8.6%; 95% CI: −16.9% to −0.4%) were predictive of lower retention rates. The finding that median cohort follow-up of ≤12 months is associated with lower 12 month retention rates again suggests that while we did not observe a strong trend towards studies with longer duration reporting higher retention rates at comparable time points, some bias may exist.

**Figure 4:**
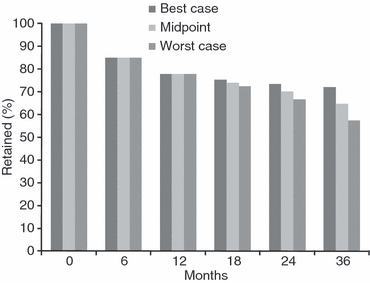
Projected Retention Rates Over Time in Antiretroviral Therapy Programs in Sub-Saharan Africa Using Varying Assumptions*. *Best-case scenario uses last observation carried forward and assumes no additional attrition after the last time point reported to. Worst-case scenario assumes a continued linear decline from the last time point reported to through 36 months. The mid-point scenario is the average of the best and worst-case scenarios. Studies are weighted by their sample size.

To project the potential paths of retention over time, we conducted three analyses to extrapolate what would have happened to retention rates at later time points for cohorts reporting only to earlier time points (Figure 4). The first set of bars shows the best case scenario, in which the latest observation is carried forward with no further attrition, while the last set of bars shows a worst case scenario which assumes that retention continued at a linear rate. There was little variation in the estimates through 24 months as the retention midpoint between the two cases is 70.0% (best-case–worst-case range: 66.7%–73.3%). By 36 months, the retention midpoint between the two cases is 64.8% (best case-worst case range: 57.5%–72.1%).

## Discussion

The global response to scaling up access to antiretroviral therapy in resource-limited settings has been rapid and dramatic and represents one of the largest public health successes in history. By the end of 2008, more than 4 million HIV infected patients had been initiated on life saving ART (Souteyrand *et al.* 2009). Even before the global economic crisis, however, the very large number of patients seeking treatment had begun to stretch resources and budgets. Now, as donors and governments face new pressure to reduce spending, difficult choices may have to be made about whether to use limited funds to initiate new patients on ART or to target funds towards keeping those already on ART alive and in care.

Our findings document both the difficulties in retaining patients in care for lifelong treatment, but also the progress being made in raising overall retention rates. Our analysis of data from more than 22 500 patients initiated on ART in sub-Saharan Africa shows that in the period since our previous analysis, overall retention in ART programs 3 years after initiating patients on treatment averaged roughly 70% using a random effects meta-analysis of reported results. This finding, while not ideal, is notable in that most of the attrition occurred in the first 2 years on treatment, when both mortality (Lawn *et al.* 2008) and loss to follow-up are known to be high. After the first 24 months, attrition averaged about 5% per year.

The retention rate at 24 months we estimated in this analysis, 76% using meta-analysis, was higher than that estimated in our earlier review, which only analysed studies through 2 years. In that analysis we estimated 62% retention by 2 years. That analysis did not use a meta-analysis approach as was performed here but instead used a weighted average approach. This suggests that overall attrition by 2 years may be slowing as experience of scaling up ART accumulates and treatment programs mature and are better able to track patients. The greater attention to measuring and understanding loss to follow-up that is reflected in the large number of publications on this topic in recent years may be paying off as programs invest in interventions to track missing patients and return them to care.

We found substantial variation in retention rates reported over time but few predictors of overall retention that explain these differences and provide guidance on what determines attrition rates. While we were only able to estimate predictors of attrition in the first year after treatment initiation, we found that low median CD4 count and having fewer females as part of the cohort were predictive of higher attrition. Programmes with low median CD4 count would be expected to have more overall attrition because of mortality. As ART programmes expand, initiating patients with higher CD4 counts could lead to both improved outcomes on treatment as well as less overall attrition. Indeed, while our current analysis showed a very similar median initiating CD4 count to our previous analysis (128 in the current analysis *vs.* 132 in the previous analysis), others have found an increasing starting CD4 count over time (Keiser *et al.* 2008). Our analysis suggests that any such increases in baseline CD4 count may also be associated with increases in retention.

Loss to follow-up constituted the highest contributor to overall attrition, as was the case in our previous report. As the number of facilities offering ART expands, more of the patients reported as lost to follow-up may in fact have transferred informally to another facility. In our analysis, among studies which reported any transfers, about 7% of all patients who initiated care transferred to another facility. While most countries find it difficult to track patients from one facility to another, developing reliable referral systems that document transfers is essential to evaluating overall programmatic effectiveness.

For those patients who are lost but do not seek care elsewhere, mortality is expected to be high (Mocroft *et al.* 1997; Morgan *et al.* 2002; Badri *et al.* 2006). Brinkhof *et al.* (2009) conducted a systematic review of outcomes among patients lost from treatment programmes and estimated that about 40% (95% CI: 33%–48%) of LTFU patients had died, with much of the mortality occurring in the first 6 months after being lost to follow-up. Making efforts to get these patients back into care is important to the overall success of ART programmes, and developing ways to track and locate lost patients is essential to proper programme evaluation (Bisson *et al.* 2007). To further evaluate programmes, when available, method such as cross referencing with vital registration systems (Anglaret *et al.* 2004; Fairall *et al.* 2008) or adjusting mortality estimates statistically (Geng *et al.* 2008; Yiannoutsos *et al.* 2008; Fox *et al.* 2010) should allow for better estimates of programme impact as well as appropriate targeting of resources towards patient retention.

Our findings should be interpreted in the light of several limitations. First, we used data collected and reported by ART programs. Some misclassification of treatment outcomes likely existed, although we have little reason to believe these misclassifications would be anything more than random. Second, we integrated information from studies that reported at many different time points, and not all reported at a time point we were interested in. In these cases we applied the retention rate to the nearest time point, but this may have caused some bias in our overall estimates. Third, we were not able to determine whether the definition of LTFU used in specific studies influenced overall retention rates because of the variability in definitions, which made it impossible in many cases to determine how long patients were absent from the clinic before being classified as lost.

Fourth, systematic reviews can be subject to publication bias. Programmes managing cohorts with higher attrition rates might be less interested in publishing their results, and this would likely lead to an underestimation of overall attrition. At the same time, better-resourced programs may be both better able to retain patients in care and to conduct and publish research, particularly long-term analyses requiring consistent data collection and strong data analysis capacity. Both of these phenomena would lead to the bias that we found in our previous analysis, in which cohorts with higher attrition rates are more likely to publish outcomes only at earlier time points. We saw some evidence for this in the current analysis, as the average 6-month retention rates were lowest for studies that only reported to 6 months compared to the average 6-month retention rates from cohorts also reporting to later time periods. Our sensitivity analysis shows that if cohorts reporting only to earlier time points had continued along at the same rate of attrition as they reported at those earlier points, overall retention would be as low as 67% at 24 months and as low as 58% by the end of 36 months. While this sensitivity analysis may overestimate attrition, even our midpoint scenario (averaging the best- and worst- case scenarios) showed that overall attrition by 36 months is likely underestimated when using only published information.

Finally, our data were not extracted in duplicate as is recommended by the Cochrane Collaboration. We instead used single data extraction. This could have resulted in some studies being missed that potentially could have met the inclusion criteria.

In conclusion, we found that overall attrition by 24 months has likely decreased slightly since our earlier report, with overall retention by 24 months averaging 70%–77% and overall retention by 36 months averaging 65%–72%. Programmes initiating patients at lower CD4 counts also had higher rates of attrition than those initiating patients with higher CD4 counts. Active tracing of lost patients to return them to care and determine their vital status if not returned should be prioritized so as to keep overall retention high and to appropriately evaluate treatment programmes.
